# Coarse-resolution Ecology of Etiological Agent, Vector, and Reservoirs of Zoonotic Cutaneous Leishmaniasis in Libya

**DOI:** 10.1371/journal.pntd.0004381

**Published:** 2016-02-10

**Authors:** Abdallah M. Samy, Badereddin B. Annajar, Mostafa Ramadhan Dokhan, Samia Boussaa, A. Townsend Peterson

**Affiliations:** 1 Biodiversity Institute and the Department of Ecology and Evolutionary Biology, University of Kansas, Lawrence, Kansas, United States of America; 2 Entomology Department, Faculty of Science, Ain Shams University, Cairo, Egypt; 3 Libyan National Centre for Disease Control (NCDC), Tripoli, Libya; 4 Department of Zoology, Faculty of Science (Sabratha), University of Zawia, Zawia, Libya; 5 Laboratory of Ecology and Environment (URAC 32, CNRST; ERACNERS 06), Faculty of Sciences Semlalia, Cadi Ayyad University, Marrakesh, Morocco; 6 Institut Supérieur des Professions Infirmières et des Techniques de Santé (ISPITS), Ministry of Health, Marrakesh, Morocco; Hitit University, Faculty of Medicine, TURKEY

## Abstract

Cutaneous leishmaniasis ranks among the tropical diseases least known and most neglected in Libya. World Health Organization reports recognized associations of *Phlebotomus papatasi*, *Psammomys obesus*, and *Meriones* spp., with transmission of zoonotic cutaneous leishmaniasis (ZCL; caused by *Leishmania major*) across Libya. Here, we map risk of ZCL infection based on occurrence records of *L*. *major*, *P*. *papatasi*, and four potential animal reservoirs (*Meriones libycus*, *Meriones shawi*, *Psammomys obesus*, and *Gerbillus gerbillus*). Ecological niche models identified limited risk areas for ZCL across the northern coast of the country; most species associated with ZCL transmission were confined to this same region, but some had ranges extending to central Libya. All ENM predictions were significant based on partial ROC tests. As a further evaluation of *L*. *major* ENM predictions, we compared predictions with 98 additional independent records provided by the Libyan National Centre for Disease Control (NCDC); all of these records fell inside the belt predicted as suitable for ZCL. We tested ecological niche similarity among vector, parasite, and reservoir species and could not reject any null hypotheses of niche similarity. Finally, we tested among possible combinations of vector and reservoir that could predict all recent human ZCL cases reported by NCDC; only three combinations could anticipate the distribution of human cases across the country.

## Author Summary

Zoonotic cutaneous leishmaniasis (ZCL) represents a major public health problem in North Africa where *Leishmania major* is the potential etiological agent associated with all ZCL cases. In many countries across North Africa, *L*. *major* is transmitted by the sand fly *Phlebotomus papatasi*, with rodents as likely reservoir hosts. In Libya, ZCL cases are underestimated for lack of reporting, insufficient information about the distribution of ZCL, and interactions between local environmental conditions and different disease components. This situation worsened with recent political and socio-economic changes in the country, with expansion and rapid increases in numbers of cases across the country. For management and planning of leishmaniasis control, predicting the potential geographic distribution of risk of infection with the disease is important to guide such programs. We introduced ecological niche model as a tool for risk-mapping of both ZCL cases and distributions of associated species. Our models were able to anticipate areas of highest risk with statistical significance, lending confidence that they were successful in identifying areas of transmission risk.

## Introduction

Leishmaniasis remains one of the major public health problems in the Mediterranean Basin. In Libya, two forms of leishmaniasis occur: visceral leishmaniasis (VL), and cutaneous leishmaniasis (CL). VL has been reported in the country since 1904; however, little information is available on leishmaniasis epidemiology as regards the insect vector species and vertebrate reservoirs involved in transmission [1–3]. VL was identified from northeastern Libya and southern Saharan and sub-Saharan areas [1,4,5]. CL is most prevalent in the northwestern part of the country [2,6,7]. CL is caused by two species of *Leishmania*: *Leishmania major* Yakimoff & Schokhor, 1914 and *L*. *tropica* Wright, 1903 (Kinetoplastida: Trypanosomatidae). *Leishmania major* is the etiological agent of zoonotic CL (ZCL), where the parasite is thought to circulate in small-mammal reservoirs (*Meriones libycus* Lichtenstein, 1823 (Rodentia: Muridae), *Gerbillus gerbillus* Olivier, 1801 (Rodentia: Muridae), *Psammomys obesus* Cretzschmar, 1828 (Rodentia: Muridae), *M*. *shawi* Duvernoy, 1842 (Rodentia: Muridae)) and is transmitted by the sand fly *Phlebotomus papatasi* (Scopoli), 1786 (Diptera: Psychodidae) [2,7,8]. *Leishmania tropica* is the causative organism for anthroponotic CL (ACL); zoonotic foci have also been reported from rock hyrax in Kenya, and Israel [9,10], and gerbil in Egypt [11], where the disease is transmitted by the sand fly *P*. *sergenti* Parrot, 1917 (Diptera: Psychodidae) [10,11].

In Libya, seasonal wadis provide potential suitable conditions of climate and vegetation for vertebrate populations to maintain transmission [12]. The sand fly *P*. *papatasi* has a wide geographic distribution, from northern Africa to India [13]; it is considered as a proven vector of ZCL in North Africa [11]. In most field surveys in the country, *P*. *papatasi* and *P*. *sergenti* were the most abundant species [7,14,15]; however, *P*. *papatasi* was most frequent in the northern part of the country.

Recently, the World Health Organization identified four possible transmission systems of ZCL, based on associated mammal reservoirs: *Ps*. *obesus*, *Meriones* spp., *Rhombomys opimus* Lichtenstein, 1823 (Rodentia: Muridae), and *Mastomys* spp. Thomas, 1915 (Rodentia: Muridae) [16]. Limited epidemiological studies have been carried out in the country to characterize the roles of several species of reservoir hosts in maintaining CL in Libya. *Leishmania major* was identified from *Meriones libycus* [12], and *M*. *shawi* [16] in endemic areas of the northwestern part of the country. Early studies revealed *Ps*. *obesus* as the potential natural reservoir host of *L*. *major* in many North African countries including Libya [12,17]; *Ps*. *obesus* was most prevalent along wadi edges from Sahara to the Middle East, where high density of this species is associated with abundant vegetation and halophilic plants [17]. *Meriones* spp. are thought to play an important role in ZCL outbreaks by maintaining the parasite in nature in the long term. *Meriones shawi* and *M*. *libycus* have been found repeatedly to be naturally infected with *L*. *major* in Libya [12], Tunisia [18–20], Morocco [21], and Algeria [22].

Most ZCL outbreaks in North African countries have been tied to epidemiological modifications and environmental changes [23,24], highlighting the importance of understanding the epidemiology of ZCL in this region. This study represents a first effort to understand the ecology and geography of ZCL using remote-sensing data across Libya to predict ZCL risk areas. We used ecological niche modeling approaches to identify the distribution of sand fly vector species, mammal reservoirs, and the pathogens to test their patterns of overlap in environmental space, which illuminate details of the local ZCL cycle in Libya where these species coexist.

## Materials and Methods

### Study area

Libya is situated in North Africa on the Mediterranean coast between Egypt and Tunisia. The country lies between 18 and 33° N latitude and 8 and 25° E longitude. Dominant climate conditions include hot-summer Mediterranean and hot desert climates [25]: coastal lowlands have very hot summers and mild winters, while the desert interior has long, hot summers and high diurnal temperature ranges, with very dry conditions. Precipitation declines rapidly to the interior with distance from the coast. Libya lacks large rivers and streams, and extended droughts are frequent; however, the government has constructed a network of dams for water management [26].

### Input data

Based on the leishmaniasis surveys in Libya, ZCL is endemic in the northwestern regions of the country. We collected records for all organisms involved in the ZCL transmission cycle including the pathogen (*L*. *major*), vector (*P*. *papatasi*), and potential mammal reservoirs (*Ps*. *obesus*, *M*. *libycus*, *M*. *shawi*, *G*. *gerbillus*). We retrieved vector and pathogen data from our own surveillance, and the PubMed database using keywords of species’ names and Libya. When *L*. *major* was identified at the coarser district level (e.g. [2]), NCDC provided details for the exact locations of these cases for the purpose of this study. *Leishmania major* records based on clinical features only were excluded from analysis to avoid possible diagnostic errors in species identification; we included all records identified rigorously by either zymodeme analysis (i.e. MON-25) of 16 enzymatic loci [27] or restriction fragment length polymorphisms of the ribosomal internal transcribed spacer 1 (ITS1) region [2]. Host and vector species included in the study were identified by reference to previously published morphological keys [28–30]. Data were included if a geographic reference was linked to any of the six species (geographic coordinates or textual descriptions). Other records of *Ps*. *obesus*, *M*. *libycus*, *M*. *shawi*, and *G*. *gerbillus* were obtained from the Global Biodiversity Information Facility (www.gbif.org), VertNet (http://www.vertnet.org/), and our own field surveillances across the country. When geographic references were textual in nature, we assigned longitude-latitude coordinates via reference to Google Earth (https://www.google.com/earth/). All occurrence data were filtered to eliminate duplicate records and longitude-latitude coordinates falling from outside Libya.

Environmental data sets by which to characterize environmental landscapes across Libya were obtained from three sources. (1) Advanced Very High Resolution Radiometer (NOAA-AVHRR) satellite imagery was obtained from the European Distributed Institute of Taxonomy (EDIT; http://bit.ly/1TDsUQM). These data comprise monthly mean Normalized Difference Vegetation Index (NDVI) coverage from 1982 to 2000, rescaled to a range of 1 to 255; we calculated mean, maximum, minimum, median, and range across the 12 monthly NDVI layers. (2) Climatic data layers representing 35 variables were obtained from global climatologies in CliMond (https://www.climond.org/; S1 File). (3) Digital elevation model were obtained from the Shuttle Radar Topography Mission (SRTM; http://srtm.usgs.gov/) at 1 km spatial resolution. All variables were resampled in ArcGIS 10.2 (Environmental Systems Resource Institute, Redlands, California) to a spatial resolution of 10 x 10' (≈20 x 20 km).

The particular environmental variables were chosen for modeling in light of their likely importance in shaping the geographic distributions of the species of interest in this study [25,31]. We selected historical NDVI and climatic data to cover the same time interval as when most records were obtained for the species. NDVI has been identified in previous epidemiological studies as an important variable by which to convey seasonality resulting from changing temperature or moisture availability, and to understand broad-scale patterns of land use and land cover and their effects on pathogen populations and transmission [32]. NDVI is significantly correlated also with details of soil conditions, including type of soil, water content, and soil moisture [33–36]. Principal components analysis (PCA) was applied to the environmental variables to reduce multicollinearity and dimensionality. We used the first 10 principal components, which summarized more than 95% of the overall variance, to summarize environmental variation across Libya.

### Ecological niche modeling

The MaxEnt algorithm [37] was used to estimate the fundamental ecological niche of the six species in this study. The fundamental ecological niche is defined as the set of environmental conditions under which a species is able to maintain populations without immigrational subsidy [38]. Correlational ecological niche models (ENMs) estimate niches by relating known occurrences to environmental values to identify conditions associated with the species presence. We calibrated ENMs within the districts where sampling was most detailed, and then transferred the model across all of Libya. MaxEnt was specified to conduct 100 bootstrapping replicates for each species. We used medians across the replicates as a final niche estimate for each species. All ENMs were converted to binary maps using a least training presence (i.e. lowest probability value of the occurrence points used in calibration of the models) thresholding approach adjusted to permit 5% omission in light of some probably erroneous records likely remaining in our data set [39].

### Model evaluation

To test the robustness of the ENMs in predicting the occurrences of the species accurately across unsampled areas of Libya, a partial receiver operating characteristic (ROC) approach was used [39]. This approach potentially allows differential weighting of omission (i.e., false negatives, leaving out actual distributional area) and commission errors (i.e., false positives, including unsuitable areas in prediction) and concentrates attention on parts of error space most relevant to niche modeling [39]. We selected 50% of the occurrence points of each species at random to test the ENMs by comparing the reduced threshold-independent area under the curve to null expectations: the dataset was bootstrapped, and probabilities obtained by direct count. AUC ratios were calculated via a software partial ROC available as a visual basic application at http://bit.ly/1JusDwz, based on 100 iterations and an *E* = 5% omission threshold.

An additional independent 98 records from the Libyan National Centre for Disease Control (NCDC) were used to test the model’s ability to predict the distribution of new ZCL cases across Libya. These samples were identified in the NCDC laboratory based on PCR protocols from previous studies (e.g. [2,11]). We checked these records to remove any occurrences matching these used in calibrating ENMs, but none coincided with those used in model calibration. We used a one-tailed cumulative binomial probability distribution that assessed the probability of obtaining the observed level of correct prediction by a chance alone given the background expectation of correct predictions and based on the proportional coverage of the region by the thresholded model prediction.

### Niche breadth and overlap

Niche breadth was estimated for each species based on the inverse concentration measure in ENMTools (http://enmtools.blogspot.com/). For successful ZCL transmission, pathogen, vector, and host species should overlap spatially and ecologically [31,40]. Here, ZCL transmission requires presence of *L*. *major*, *P*. *papatasi*, and at least one of the mammal reservoir species. We used background similarity tests [41] to assess similarity between pairs of estimated niches. We first estimated the accessible area (**M**) for each species in the study [42]; the accessible area for *L*. *major* was identified based on the distribution of that species across the country, where the species occurs only in the northwestern part. **M** estimates for the other species included all or at least a subset of the northern parts of the country depending on the species’ current distributions.

To test the null hypothesis of niche similarity between each pair of niches, we used *D*-statistics and Hellinger's *I* implemented in ENMTools [41]. Niche similarity was tested with respect to all environmental variables used to develop the ENM for each species. The background similarity test is based on generating random points from across the accessible area of one species in numbers equal to the numbers of real occurrence data available for that species in the study, with 100 replicate samples, and comparing an ENM based on these “background” points to the ENM of the other species. The null hypothesis of niche similarity was rejected if the *D* or *I* values fell below the 5^th^ percentile in the random-replicate distribution of similarity values [41].

We assumed that areas could be considered as at risk of ZCL transmission when all necessary elements for transmission co-occur [40]. We used the ENMs for *P*. *papatasi* and the four candidate ZCL reservoirs to identify areas of overlap between the vector and each of the possible hosts. These grids were obtained by multiplying the binary ENM of *P*. *papatasi* with the binary grid for the host species. We used a one-tailed cumulative binomial test to assess the relationship between the areas of vector-reservoir overlap and independent leishmaniasis human case records from the NCDC.

## Results

**Fig 1:**
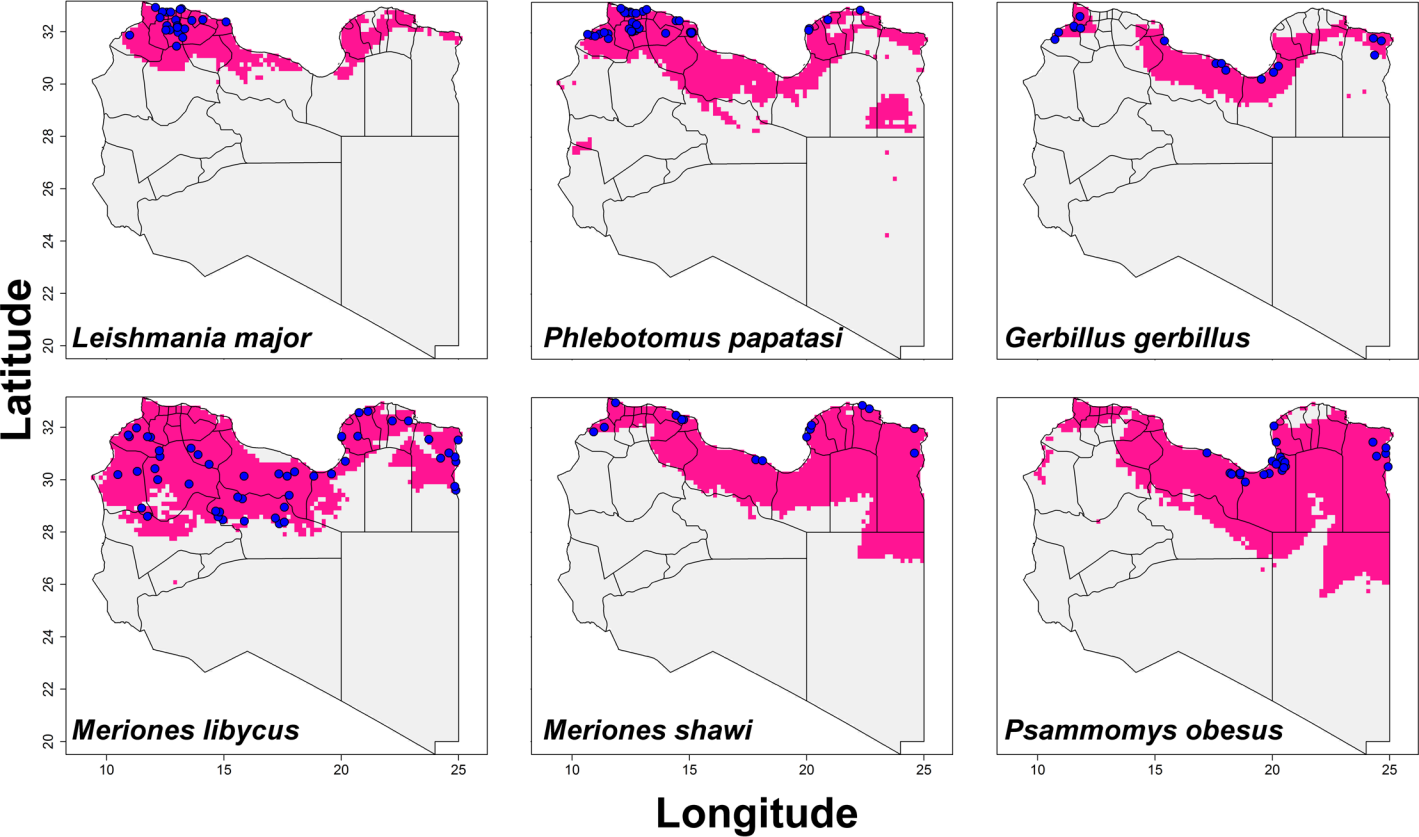
Thresholded potential distribution maps for *Leishmania major*, *Phlebotomus papatasi*, and four candidate mammal reservoir species potentially associated with the zoonotic transmission of cutaneous leishmaniasis. Models were calibrated across sampled areas (S), and transferred across all Libya. Blue points are occurrences, pink areas are modeled suitable conditions, and gray areas are unsuitable conditions.

We collected a total of 348 occurrences for *P*. *papatasi*, *L*. *major*, and four candidate reservoir species across Libya. Occurrence records are fully and openly available via Figshare repository (https://dx.doi.org/10.6084/m9.figshare.1613478). These data were concentrated along the northern coast of Libya (Fig 1). *Phlebotomus papatasi* was recorded from 84 localities, whereas *L*. *major* was characterized by 50 localities. *Meriones libycus* was the most commonly recorded mammal reservoir (104 sites) followed by *Ps*. *obesus* (48), *G*. *gerbillus* (32), and *M*. *shawi* (30). ENMs developed for these six species are illustrated in Fig 1; ENMs calibrated across the country (for comparison) are presented in the Supporting Information (S2 File).

ENMs predicted most of the species to range across the northern coast of Libya; however, three species had broader potential distributions extending south to central Libya (*M*. *libycus*, *M*. *shawi*, *Ps*. *obesus*). The ENM for *L*. *major* predicted highest suitability in a belt between 30–33° N. These areas included many western provinces (e.g., Nalut, Yafran, Nuqat Al Khams, Al Jifarah, Sabratah, Misrata, Al Marqab, Gharyan, Babratah, Az Zawiya, Tajura, Tarhunati, Bani Walid, and Sirte), but also some eastern provinces (e.g., Al Jabal Al Akhdar, Al Qubbah, Al Hizam Al Akhdar, and Ajdabiya). The potential distribution of *P*. *papatasi* extended across the northern coast of Libya, but also in a disjunct area in central east Libya. All ENMs calibrated for these species were significantly robust based on partial ROC tests, with AUC ratios uniformly above 1 (*P* < 0.001; Table 1).

**Table 1:** Results of partial ROC analysis to test statistical significance of ecological niche model predictions. A value of 1.0 is equivalent to the performance of a random classifier. These results were based on 100 bootstrap replicates, and statistical significance was assessed via bootstrapping and comparison with a random classifier ratio of 1.0.

Species	Mean	Minimum	Maximum
*Leishmania major*	1.92	1.91	1.95
*Phlebotomus papatasi*	1.94	1.93	1.98
*Gerbillus gerbillus*	1.57	1.50	1.88
*Meriones libycus*	1.56	1.35	1.89
*Psammomys obesus*	1.71	1.64	1.96
*Meriones shawi*	1.78	1.72	1.94

**Fig 2:**
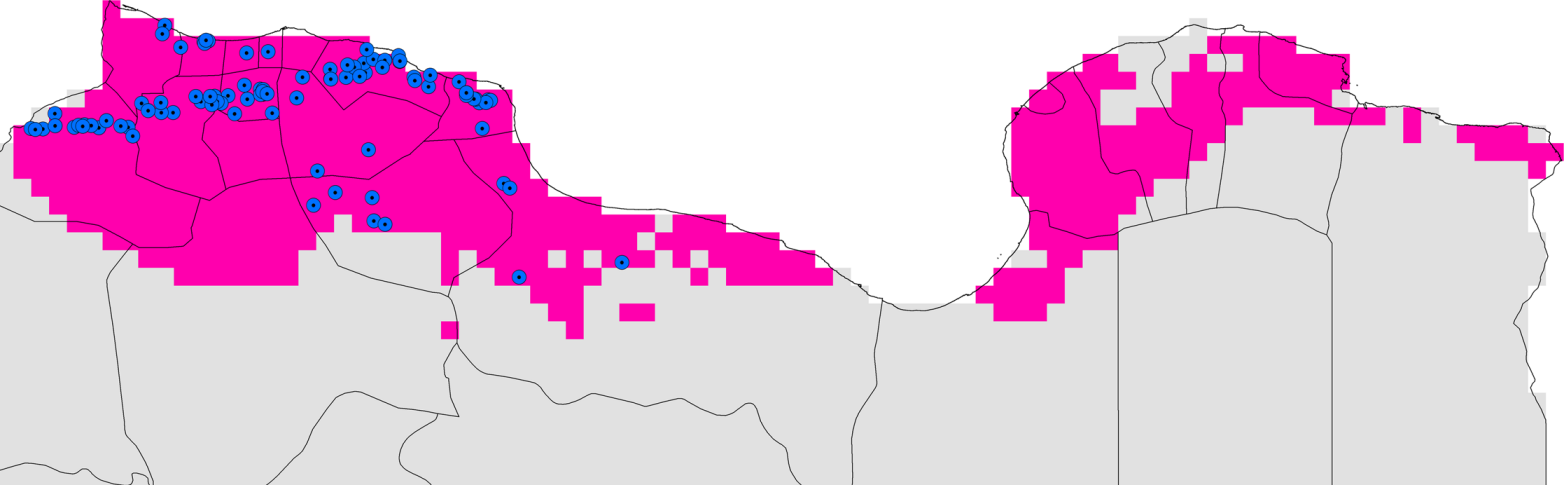
Relationship of ecological niche modeling predictions to the distribution of 98 sites with *L*. *major* cases reported by the Libyan National Centre for Disease Control in recent outbreaks across Libya. The blue dotted circle represented localities where these independent data were collected, and pink represent the belt predicted suitable for the *Leishmania major*.

In the most recent CL outbreaks across Libya, NCDC identified *L*. *major* in cases from 98 sites. The *L*. *major* ENM predicted 98 out of 98 of these additional independent data, which is statistically better than random expectations (*P* < 0.001). These additional independent data thus corroborated the *L*. *major* ENM, and the ability of that model to anticipate all recent cases of ZCL identified (Fig 2).

**Fig 3:**
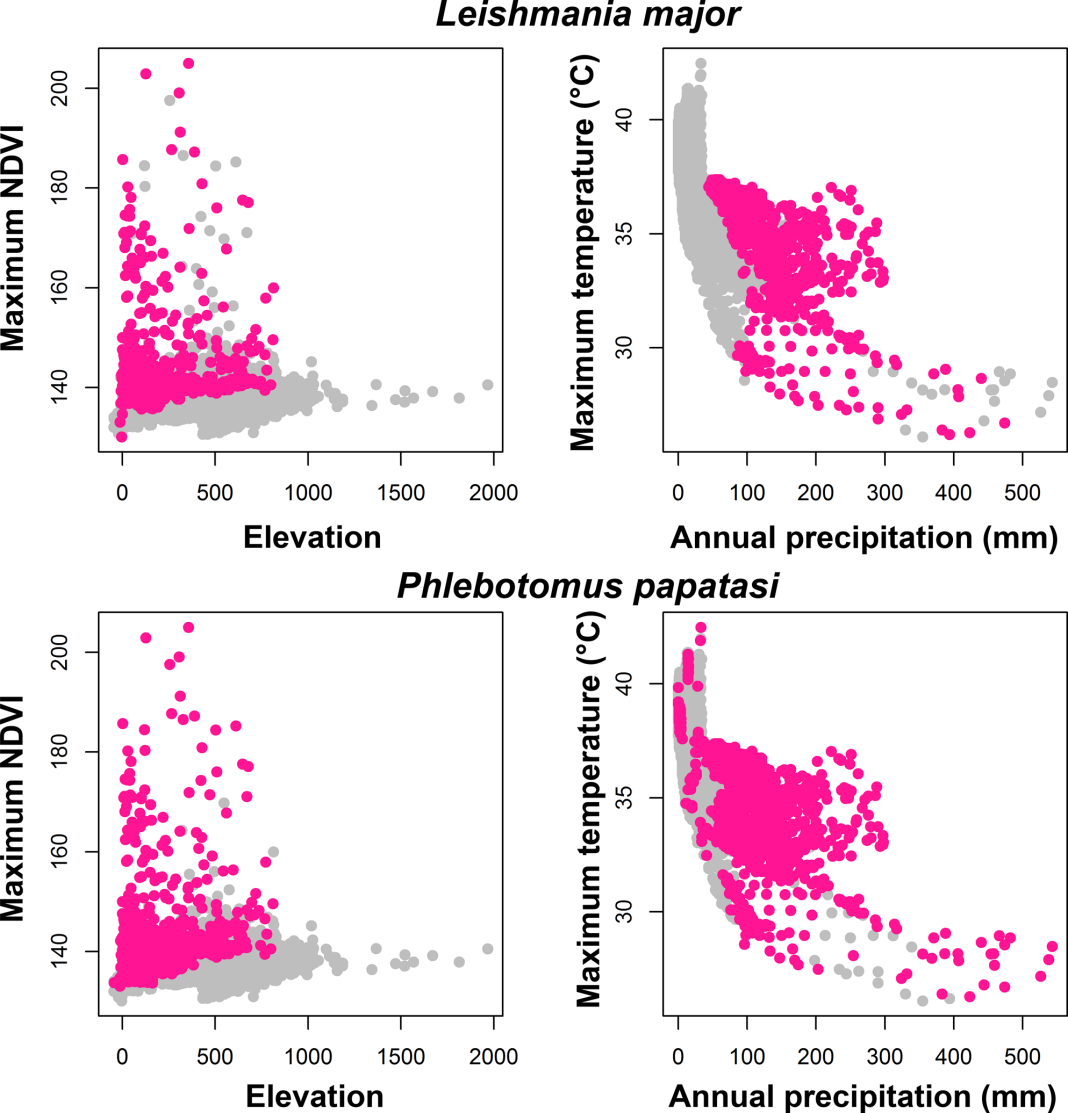
Visualization of *Leishmania major*, and *Phlebotomus papatasi* ecological niches in example dimensions. Overall set of environments available across Libya in gray; modeled suitable conditions for the species occurrences in pink. Similar visualizations of ecological niches for the potential mammal reservoir species are in the Supporting Information (S4 File).

Niche breadth was least in *L*. *major* and *P*. *papatasi*, and greater in the mammal species; indeed only *G*. *gerbillus* had niche breadth similar to *L*. *major* (S3 File). We visualized the environmental conditions where these species occur: *L*. *major* and *P*. *papatasi* were at low elevations, and mostly under a maximum temperature of 25°C–37°C (Fig 3). The other species had similar responses to environmental conditions; however, they tend to be distributed along a broader environmental range (except *G*. *gerbillus*; S4 File).

**Fig 4:**
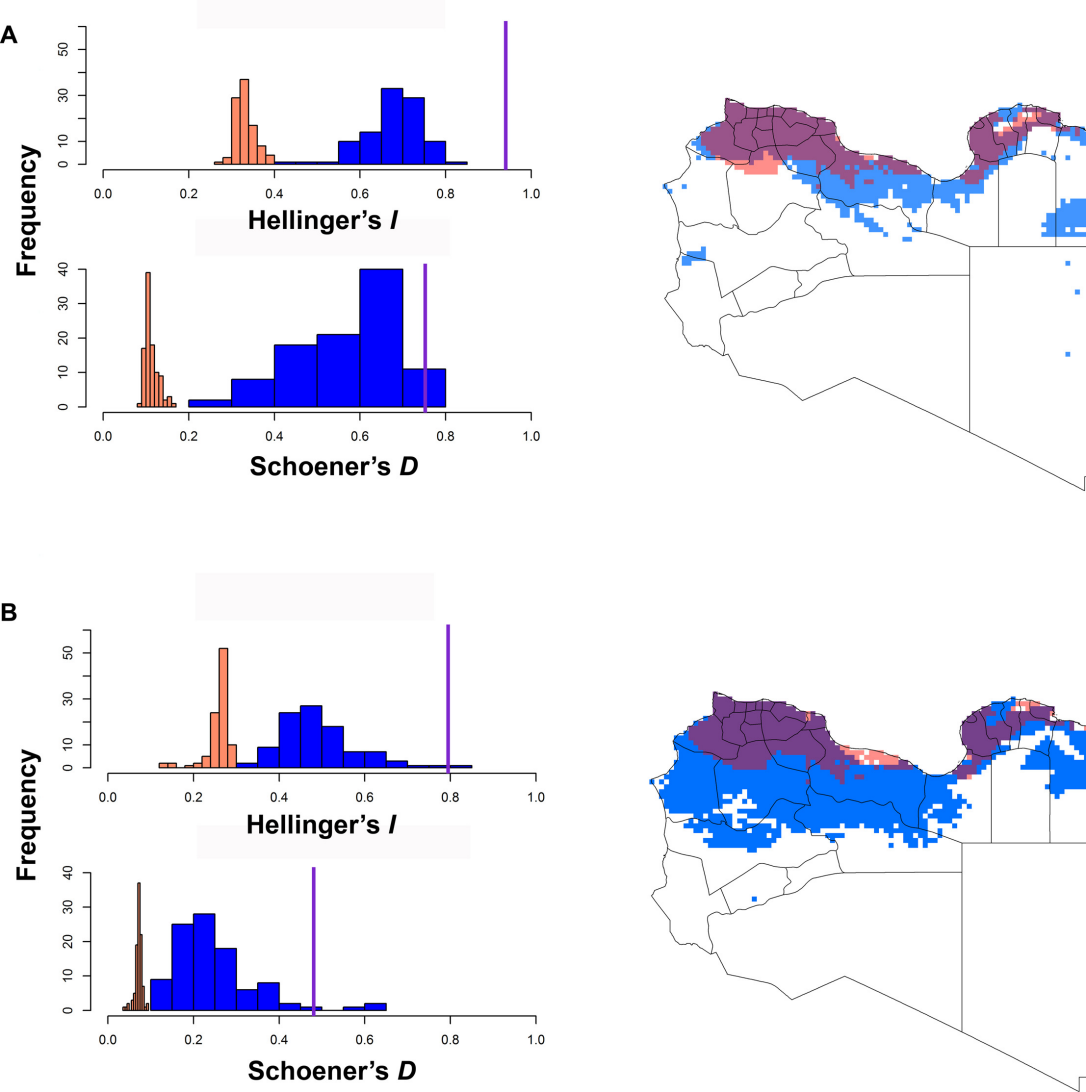
Example background similarity tests showing overall niche overlap between ecological niche models for pairs of species: (A) *Leishmania major*—*Phlebotomus papatasi* and (B) *Leishmania major* –*Meriones libycus*. The vertical purple line shows observed niche overlap, and the histograms show the distribution of the background similarity values among 100 random replicates, for the *I* and *D* similarity metrics. On the maps, red and blue shading indicates the modeled suitable areas for the two species; purple shading shows areas of overlap between the two species. Results for other species are given in the Supporting Information (S5 File).

**Fig 5:**
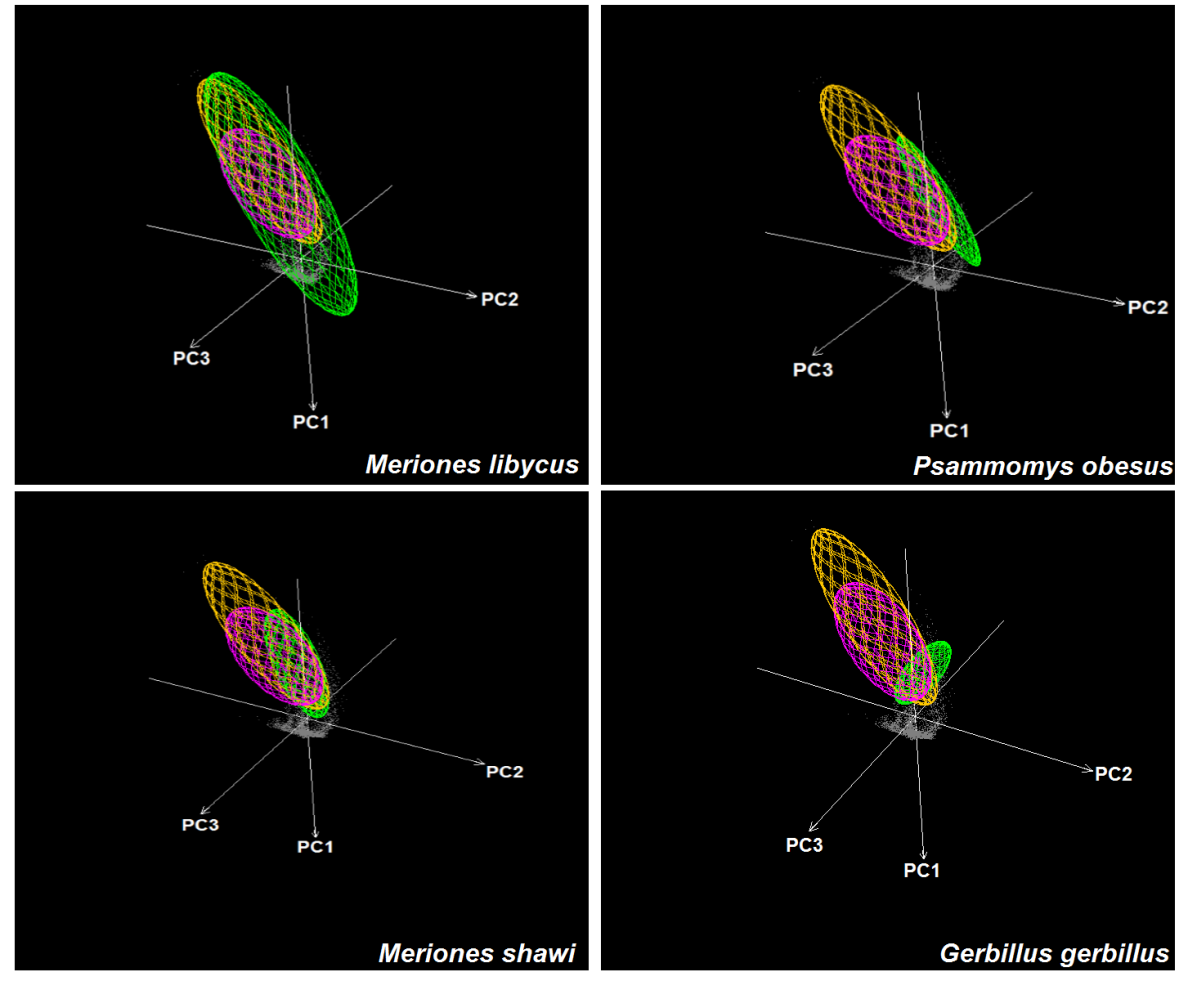
Visualization of ecological niches of *Leishmania major*, *Phlebotomus papatasi*, and animal reservoir in three environmental dimensions (PC1, PC2, and PC3). Niches are represented as minimum volume ellipsoids to illustrate the limits under which the species has been sampled. Gray shading represents environmental background, green ellipsoid represents the potential mammal reservoir, yellow is the vector *Phlebotomus papatasi*, and purple represents *Leishmania major*.

The background similarity tests comparing the ENMs of parasite, vector, and possible reservoirs were uniformly unable to reject the null hypothesis of niche similarity between these species (*P* > 0.05; Fig 4 & S5 File). This result indicates the niche estimate for *L*. *major* could not be distinguished from those of the vector or the four potential reservoirs. We used NicheA to visualize overall overlap between the species based on three dimensions of PCAs (Fig 5), which revealed broad overlap in environmental conditions used by six species.

**Fig 6:**
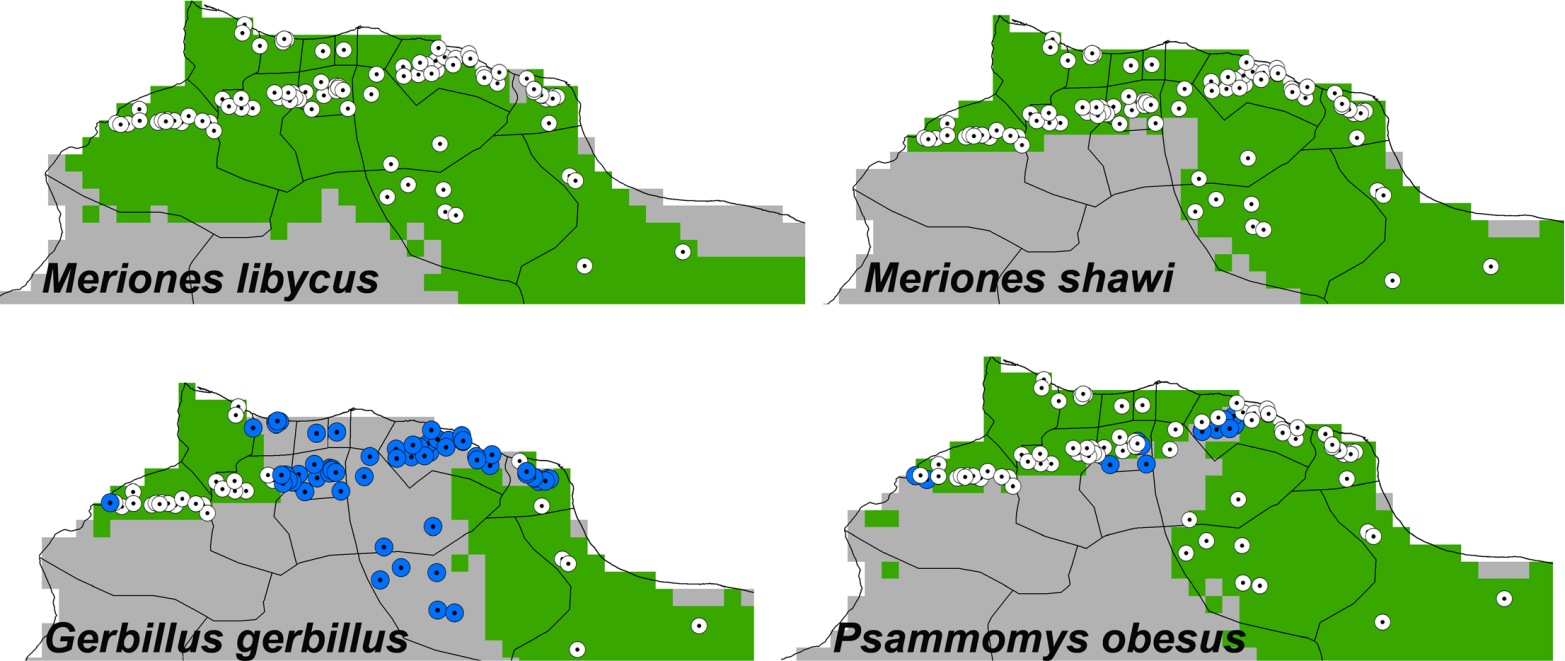
Relationship of additional independent human case records to the areas where pairs of vector *Phlebotomus papatasi* and mammal reservoir species can occur. Green areas are areas of overlap between *P*. *papatasi* and each of the potential mammal reservoirs; white dotted circle represent localities where human cases were predicted successfully; blue dotted circles indicate case records not predicted successfully by the model combination.

Finally, we combined the modeled distribution of the vector *P*. *papatasi* with those of each of the potential reservoirs as hypotheses of system that could support zoonotic transmission of CL across Libya (Fig 6). Results revealed that *P*. *papatasi*-*M*. *libycus*, *P*. *papatasi*-*M*. *shawi*, and *P*. *papatasi-Ps*. *obesus* systems predicted recent ZCL well (*P* < 0.01); the first two predicted 100% of the cases reported to the NCDC, but *Ps*. *obesus* identified only 85.7% of these cases (84 out of 98). The *P*. *papatasi*-*G*. *gerbillus* map was able to predict only 29 of 98 records, not better than null expectations (*P* > 0.05).

## Discussion

Numerous recent studies have attempted to map potential distributions of key species involved in leishmaniasis transmission in several countries in Europe and the Americas [43,44]. Africa, however, has seen only a few efforts to map vector populations [31,45–47]. Libya sees many CL cases [3]; for example, 6284 cases were identified there in 2006 alone (S6 File). CL case rates are still underestimated owing to inefficient infrastructure for early notifications of cases, and lack of public awareness among doctors and patients [3]. For the national control program to be successful, all organisms associated with leishmaniasis transmission should be identified and understood in detail (i.e. vectors, reservoirs, and pathogens).

We developed this mapping exercise across Libya for several reasons. (1) Most prominently, we wished to map the potential distribution of ZCL cases across the country. (2) We strove to map the potential distribution of 5 other organisms potentially associated with the disease’s dynamics in Libya. (3) We wished to test niche similarity among the set of species involved. Finally, (4) we tested the possible reservoir-vector combinations that could allow better prediction of ZCL cases. All of these analyses will help to understand the disease risk areas across the country and guide possible control programs.

Our models identified risk areas across both the western and eastern portions of the north coast of the country. Although all previous studies in Libya had found CL cases only in the western provinces, some recent reports have provided evidence of CL occurrence in eastern sites as well (e.g. Ajdabiya, and Al Jabal Al Akhdar; [48]). Although this report [48] is the only one to place CL at these sites, most CL surveillance has concentrated in western Libya [2,6,7], so this results is perhaps expected. Our ENMs found suitability of both regions for ZCL transmission, benefitting from higher-resolution environmental data, and consideration of areas that were sampled and accessible to each species [42,49].

The risk of ZCL transmission in North Africa appears to be determined by the joint dynamics of vectors and mammal reservoir populations [16]. When we visualized the environmental conditions suitable for the species examined in this study, they were most prevalent in a maximum temperature range of 25°C–37°C, similar to other recent reports across North Africa and Middle East [50–52]. These latter studies reported that *P*. *papatasi* was abundant in semi-arid and arid steppe zones, and that low and high temperatures are key in limiting its distribution and activity [50–52]. For example, *P*. *papatasi* in Morocco was less active at temperatures of 11–20°C and 37–40°C [52]. The distributional patterns of *L*. *major* and *P*. *papatasi* estimated in this study concords with these latter reports [51,52]. Northern coastal regions of Libya are characterized by a Mediterranean climate, whereas the rest of the country has hot, dry desert climates that are unfavorable for these species, with maximum summer temperatures over 40°C apparently. Previous studies have shown that water is a major limiting factor for sand flies and for leishmaniasis abundance and spread, respectively [53]. *Phlebotomus papatasi* cannot tolerate the extreme conditions of temperature and low humidity associated with the rare rainfall in the south, although the species is well established in other deserts where conditions are more mesic (e.g., Negev Desert [53]). Our study identified an interesting prediction of the presence of suitable environmental conditions in central Libya, associated with construction of new water resources [26] and raised concerns for changes in the eco-epidemiology of leishmaniasis across the country as water resources (S7 File) and agricultural activities are established in southern parts of the country. These important anthropogenic changes will be key factors in affecting distributions of vectors and reservoir hosts of ZCL across Libya; for example, in other studies in the region, soil moisture was an important variable in determining vector and reservoir abundance [54,55]; anthropogenic disturbance was also identified as favoring conditions for vector and larger host populations in Israel [56]. The effects of these two factors may be reflected among some of environmental variables included in our study, but their absence in explicit terms still marks a limitation to our study; a more detailed picture of ZCL transmission risk in the region will need to consider their possible effects on long-term sand fly and rodent abundances.

Most recent ZCL cases occurred at relatively low elevations; the areas near Al Jabal Al Gharbi alone accounted for most cases (S7 File) [2,57]. Similar observations were reported for *L*. *major*, *P*. *papatasi*, and wild mammals in Morocco [58]. Elevation and temperature are not the only factors influencing the distribution of ZCL cases: precipitation has also been shown to play a role [45]. Low-elevation northern areas, where *L*. *major* and *P*. *papatasi* species occur in high densities, are characterized by the highest precipitation in the country [45].

Although testing niche similarity among species was unable to distinguish among hypotheses of ZCL hosts, as was possible in our previous analyses in Egypt [31], our analyses of possible species combinations excluded *G*. *gerbillus* as a main reservoir across Libya. In our previous analysis in Egypt, however, we found marked niche similarity between *P*. *papatasi* and *L*. *major*, but none between *L*. *major* and *P*. *sergenti* in terms of geographic distribution and ecological niche [31], supporting the idea that carefully constructed ENMs are able to predict disease risk based on models of vectors and reservoir hosts in a complex transmission system like leishmaniasis. In this study, 85.7% of cases were predicted successfully, focusing on areas where *Ps*. *obesus* co-occurred with the vector. WHO had reported that *Ps*. *obesus* was likely the main reservoir of ZCL in Libya [16]; however, our results more strongly supported the two *Meriones* spp.–*P*. *papatasi* system, which were able to anticipate all recent human cases. Evidence for this association has also been found in the form of high infection rates with *L*. *major* in *Meriones tristrami* in the most recent ZCL foci documented in Israel [59]; these observations provide mounting evidence that jird play a major role in disease transmission across the region. This study took Libya as a target population for illuminating the identity and distribution of reservoir hosts in the complex ZCL cycle. Indeed, simply the definition of “reservoir host” remains unresolved [60–62]; early studies defined reservoir host as the “ecological system in which the infection agent survives indefinitely” [60], but later studies focused on definition in reference to a specific target population [61]. Certainly, some confusions in reservoir definition still exist; the specification of particular target populations emphasizes the importance of geographic and ecological associations in defining reservoir hosts, which underlines the approach in this study. As a result, we urge development of similar studies regarding other target populations to examine spatial and temporal relationships of these hosts, and characterize differences in ZCL dynamics among regions.

The study of the association among these organisms in both spatial and temporal dimensions is of great added values to map the ZCL risk areas across Libya, guide the control program across the country, and provided the first detailed maps for the potential distributions of organisms associated with the zoonotic transmission cycle across Libya. An early study shed light on disease ecology and possible host-pathogen associations [63], discussing criteria of host geographic distribution, pathogen range within the host range, regional distributions of organisms in different biomes and habitats, relative prevalence of the pathogen among host subpopulations, temporal and fine-scale spatial pattern of host-pathogen dynamics, and integrative time- and place-specific predictive models. These criteria were discussed as major steps to promote understanding of pathogen-host associations in complex transmission cycles. This study applied most of these criteria to the complex ZCL cycle in Libya but we note knowledge gaps in Libya regarding the prevalence of *L*. *major* among different host subpopulations, and the dynamics and potential distribution of host and parasite at finer scales across the country. Filling these gaps as regards the disease system in Libya will promote a more detailed picture both for its ecology and for control programs.

Leishmaniasis control programs should consider our findings by applying integrated approaches to combating ZCL by considering the environmental risk factors that we have explored. That is, if a particular combination of host and vector species is necessary for leishmaniasis transmission, then strategies by which to interrupt that transmission can focus on removing the pathogen, the vector, or key hosts from the system. Such measures may be implemented via educational programs in risk areas, mass drug administration in infected communities, and host or vector control programs. Our future work will focus on possible hotspots in the less-well-known areas of the country via intensive disease surveillance and sampling of all relevant organisms. More deeply, we plan to consider socioeconomic variables in tandem with the physical environmental variables for a more universal model that links physical, biological, and human factors in this complex disease system.

## Supporting Information

S1 File Detailed description of the CliMond variables used in the model. Details on these variables are also available via https://www.climond.org/.
(PDF)

S2 File Thresholded potential distribution maps for *Leishmania major*, *Phlebotomus papatasi*, and four candidate mammal reservoir species potentially associated with the zoonotic transmission of cutaneous leishmaniasis. Models were calibrated directly across Libya. The pink areas represent modeled suitable conditions, and gray areas were modeled as unsuitable for the species.
(PDF)

S3 File Values of niche breadth for *Leishmania major*, *Phlebotomus papatasi*, and the four potential mammal reservoirs. (PDF)

S4 File Visualizations of ecological niches of four potential mammal reservoirs in two environmental dimensions. The diagram shows the overall environment available across Libya (gray), and the suitable conditions for species occurrences (pink).
(PDF)

S5 File Background similarity tests of ecological niche overlap between species. The red vertical line represent the observed niche overlap between the two ENMs in the question. The results of the background similarity tests were based on Schoener’s *D* (left column) and Hellinger’s *I* (right column) similarity metrics.
(PDF)

S6 File Total annual number of cases reported to the Libyan National Centre for Disease Control 2004–2013. These cases were reported by the local health units in each province and notified to the center for control measures based on the endemic status of each focus. These cases were identified by passive surveillance, and were not diagnosed to the species level.
(PDF)

S7 File Localities with high zoonotic cutaneous leishmaniasis incidence and water resource management across Libya. Districts with high incidence are shown in blue, and localities within each district is presented as a dotted points. The map of Libya at the top shows the distribution of areas with water resource management initiatives as blue circles.
(PDF)
