# Supplementary material for: Coarse-resolution Ecology of Etiological Agent, Vector, and Reservoirs of Zoonotic Cutaneous Leishmaniasis in Libya
Source: PLoS Negl Trop Dis. 2016 Feb 10;10(2):e0004381. doi: 10.1371/journal.pntd.0004381 (PMC4749236; doi:10.1371/journal.pntd.0004381)
Supplement: S1 File — Details on these variables are also available via https://www.climond.org/. (PDF) [file pntd.0004381.s001.pdf]

S1 File: Detailed description of the CliMond variables used in the model. Details on these variables are also available via https://www.climond.org/.

Variable Number	Variable
Bio01	Annual mean temperature (°C)
Bio02	Mean diurnal temperature range (mean(period max-min)) (°C)
Bio03	Isothermality (Bio02 ÷ Bio07)
Bio04	Temperature seasonality (C of V)
Bio05	Max temperature of warmest week (°C)
Bio06	Min temperature of coldest week (°C)
Bio07	Temperature annual range (Bio05-Bio06) (°C)
Bio08	Mean temperature of wettest quarter (°C)
Bio09	Mean temperature of driest quarter (°C)
Bio10	Mean temperature of warmest quarter (°C)
Bio11	Mean temperature of coldest quarter (°C)
Bio12	Annual precipitation (mm)
Bio13	Precipitation of wettest week (mm)
Bio14	Precipitation of driest week (mm)
Bio15	Precipitation seasonality (C of V)
Bio16	Precipitation of wettest quarter (mm)
Bio17	Precipitation of driest quarter (mm)
Bio18	Precipitation of warmest quarter (mm)
Bio19	Precipitation of coldest quarter (mm)
Bio20	Annual mean radiation (W m^{-2})
Bio21	Highest weekly radiation (W m^{-2})
Bio22	Lowest weekly radiation (W m^{-2}
Bio23	Radiation seasonality (C of V)
Bio24	Radiation of wettest quarter (W m^{-2})
Bio25	Radiation of driest quarter (W m^{-2})
Bio26	Radiation of warmest quarter (W m^{-2})
Bio27	Radiation of coldest quarter (W m^{-2})
Bio28	Annual mean moisture index
Bio29	Highest weekly moisture index
Bio30	Lowest weekly moisture index
Bio31	Moisture index seasonality (C of V)
Bio32	Mean moisture index of wettest quarter
Bio33	Mean moisture index of driest quarter
Bio34	Mean moisture index of warmest quarter
Bio35	Mean moisture index of coldest quarter
