# Supplementary material for: Coarse-resolution Ecology of Etiological Agent, Vector, and Reservoirs of Zoonotic Cutaneous Leishmaniasis in Libya
Source: PLoS Negl Trop Dis. 2016 Feb 10;10(2):e0004381. doi: 10.1371/journal.pntd.0004381 (PMC4749236; doi:10.1371/journal.pntd.0004381)

S2 File: Thresholded potential distribution maps for *Leishmania major*, *Phlebotomus papatasi*, and four candidate mammal reservoir species potentially associated with the zoonotic transmission of cutaneous leishmaniasis. Models were calibrated directly across Libya. The pink areas represent modeled suitable conditions, and gray areas were modeled as unsuitable for the species.

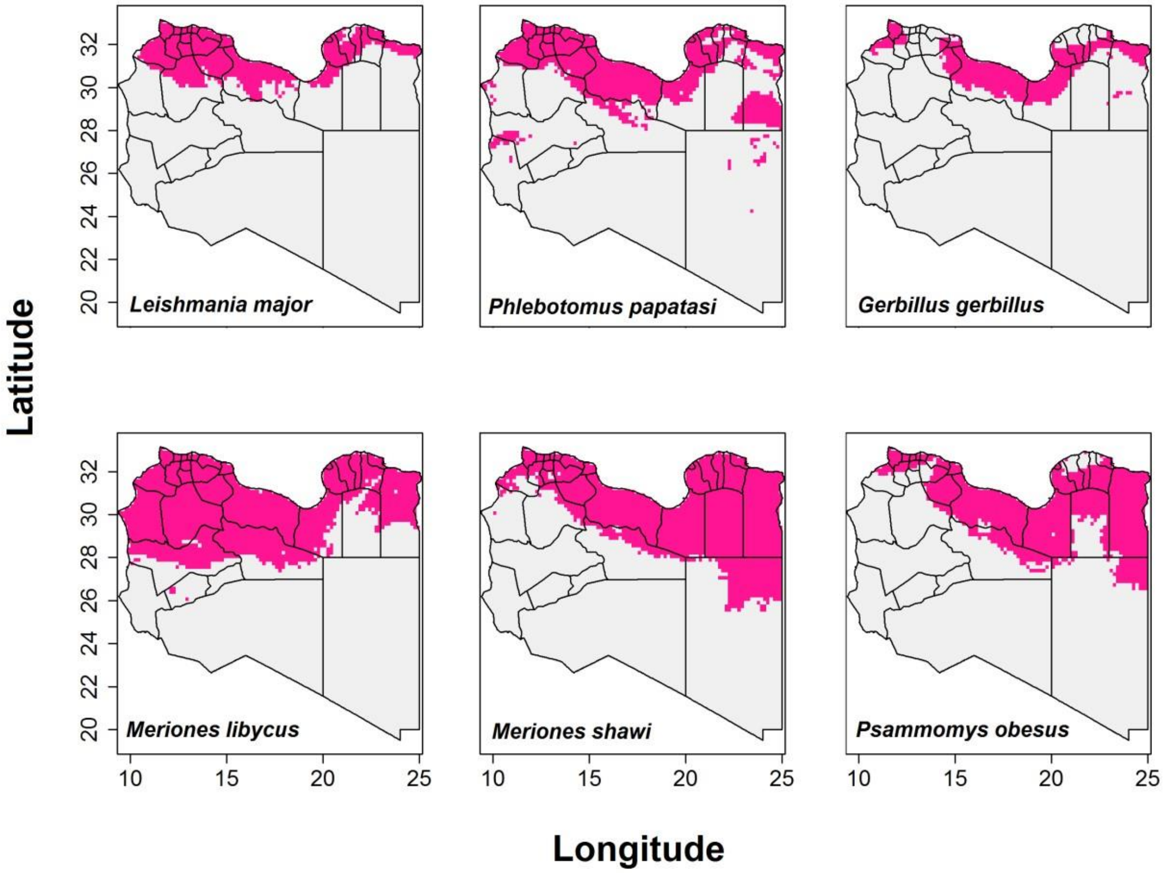

Supplement: S2 File — Models were calibrated directly across Libya. The pink areas represent modeled suitable conditions, and gray areas were modeled as unsuitable for the species. (PDF) [file pntd.0004381.s002.pdf]
