# Supplementary material for: Coarse-resolution Ecology of Etiological Agent, Vector, and Reservoirs of Zoonotic Cutaneous Leishmaniasis in Libya
Source: PLoS Negl Trop Dis. 2016 Feb 10;10(2):e0004381. doi: 10.1371/journal.pntd.0004381 (PMC4749236; doi:10.1371/journal.pntd.0004381)
Supplement: S3 File — (PDF) [file pntd.0004381.s003.pdf]

S3 File: Values of niche breadth for *Leishmania major*, *Phlebotomus papatasi*, and the four potential mammal reservoirs.

Species	Niche breadth
Leishmania major	0.097
Phlebotomus papatasi	0.165
Meriones libycus	0.283
Gerbillus gerbillus	0.092
Meriones shawi	0.246
Psammomys obesus	0.317
