# Supplementary material for: Coarse-resolution Ecology of Etiological Agent, Vector, and Reservoirs of Zoonotic Cutaneous Leishmaniasis in Libya
Source: PLoS Negl Trop Dis. 2016 Feb 10;10(2):e0004381. doi: 10.1371/journal.pntd.0004381 (PMC4749236; doi:10.1371/journal.pntd.0004381)

S4 File: Visualizations of ecological niches of four potential mammal reservoirs in two environmental dimensions. The diagram shows the overall environment available across Libya (gray), and the suitable conditions for species occurrences (pink).

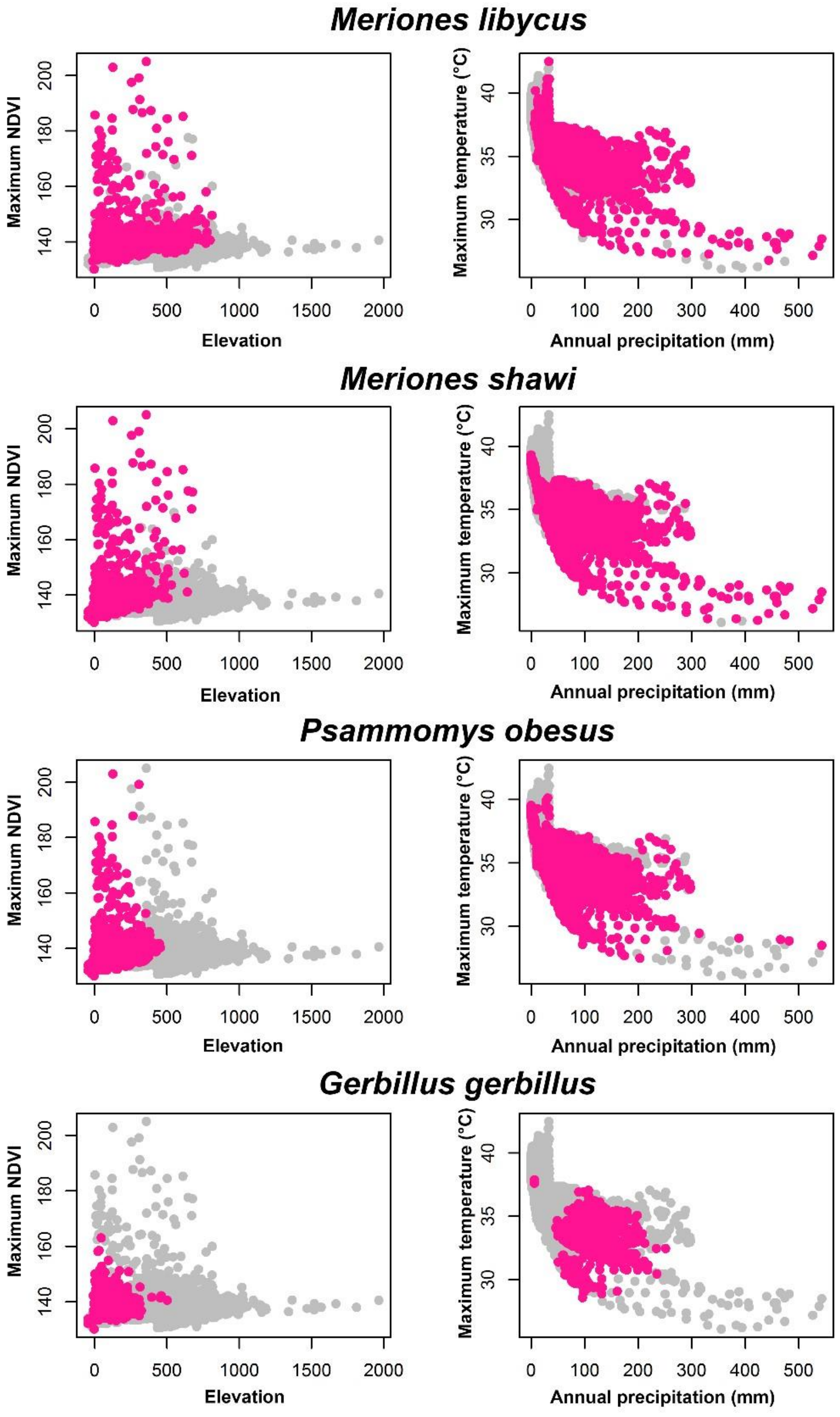

Supplement: S4 File — The diagram shows the overall environment available across Libya (gray), and the suitable conditions for species occurrences (pink). (PDF) [file pntd.0004381.s004.pdf]
