# Supplementary material for: Coarse-resolution Ecology of Etiological Agent, Vector, and Reservoirs of Zoonotic Cutaneous Leishmaniasis in Libya
Source: PLoS Negl Trop Dis. 2016 Feb 10;10(2):e0004381. doi: 10.1371/journal.pntd.0004381 (PMC4749236; doi:10.1371/journal.pntd.0004381)
Supplement: S5 File — The red vertical line represent the observed niche overlap between the two ENMs in the question. The results of the background similarity tests were based on Schoener’s D (left column) and Hellinger’s I (right column) similarity metrics. (PDF) [file pntd.0004381.s005.pdf]

S5 File: Background similarity tests of ecological niche overlap between species. The red vertical line represent the observed niche overlap between the two ENMs in the question. The results of the background similarity tests were based on Schoener's *D* (left column) and Hellinger's *I* (right column) similarity metrics.

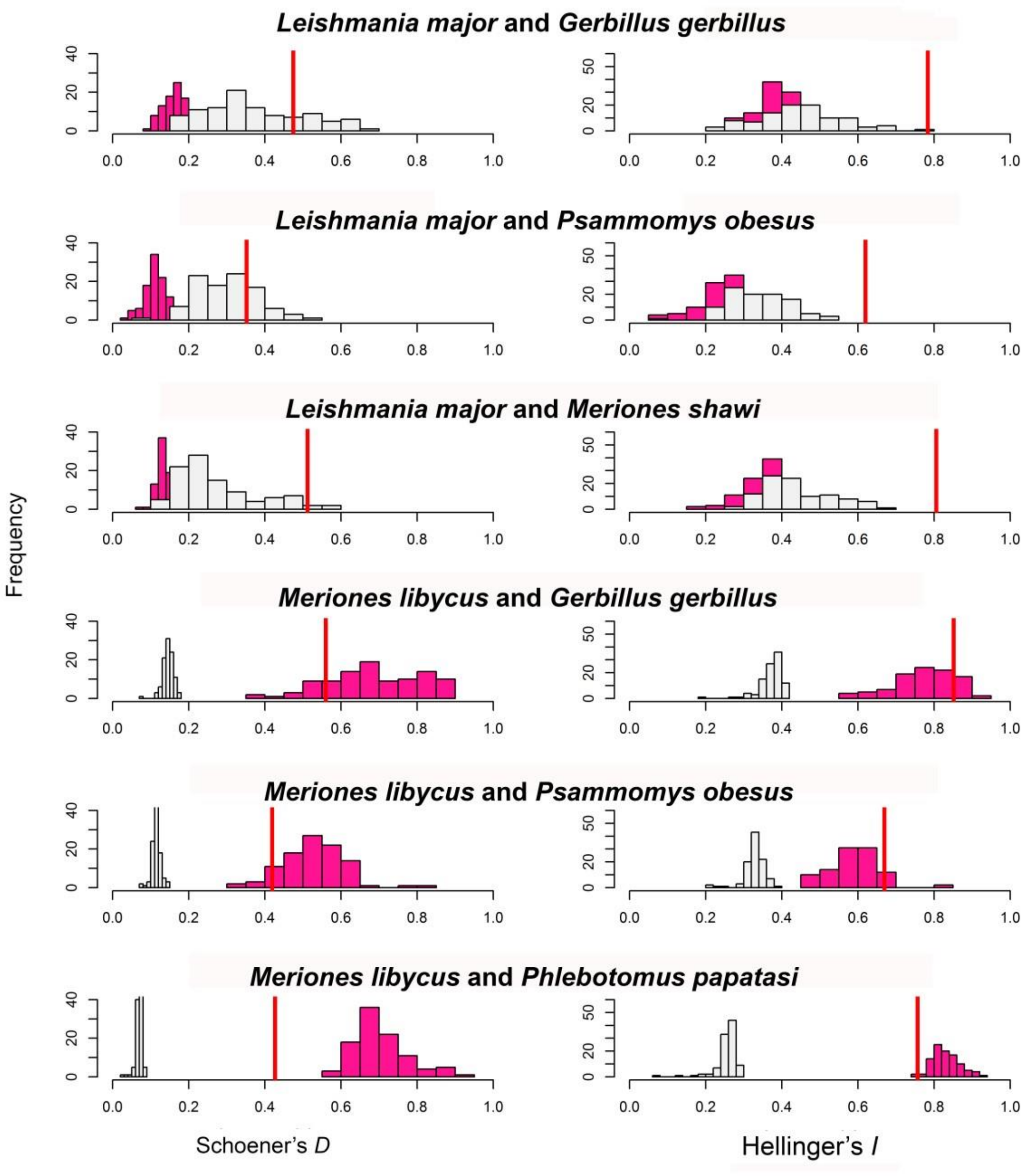

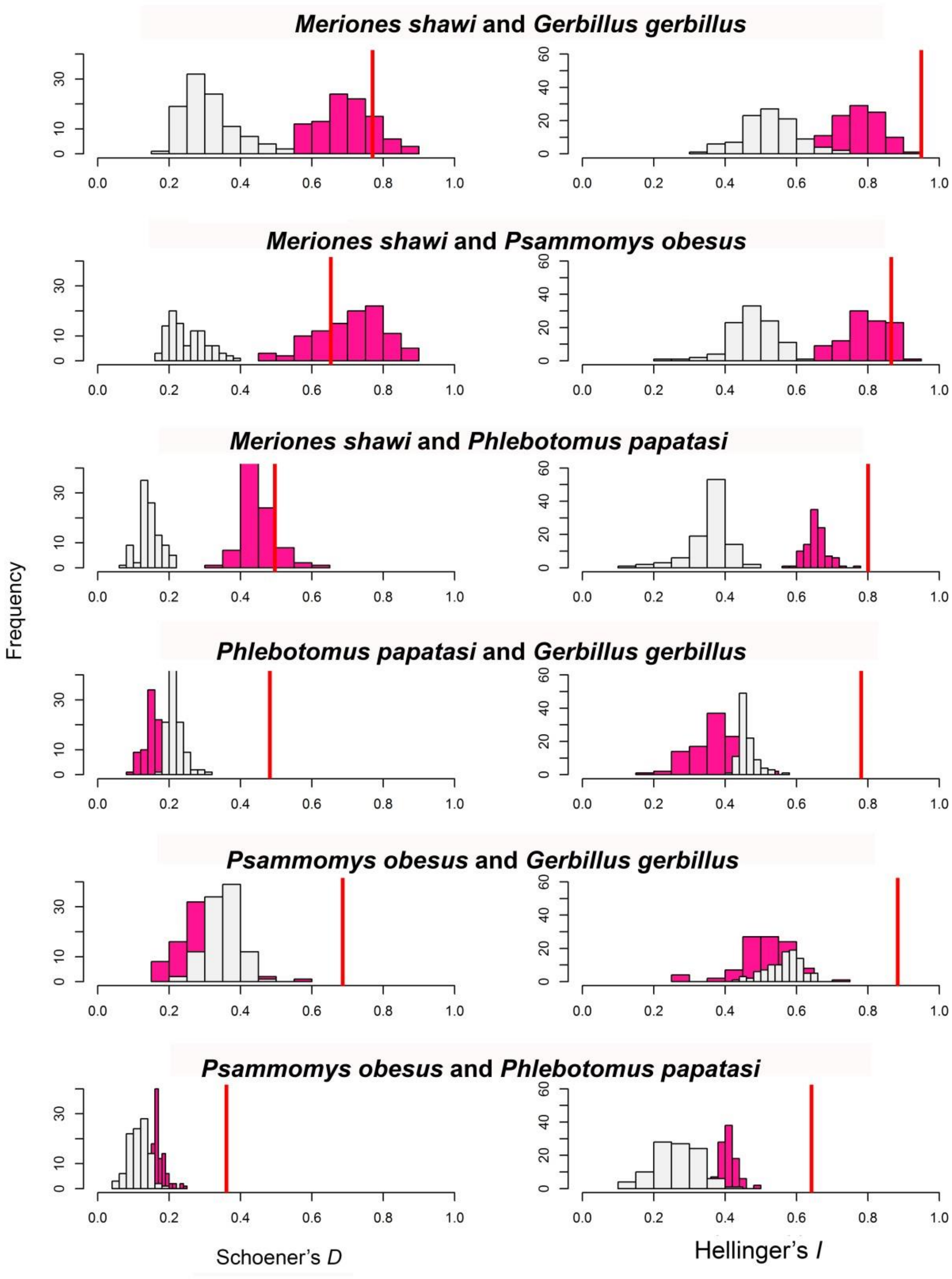

Meriones shawi and Gerbillus gerbillus
Meriones shawi and Psammomys obesus
Meriones shawi and Phlebotomus papatasi
Phlebotomus papatasi and Gerbillus gerbillus
Psammomys obesus and Gerbillus gerbillus
Psammomys obesus and Phlebotomus papatasi
Frequency
0
10
30
20
40
60
0.0
0.2
0.4
0.6
0.8
1.0
Schoener's D
Hellinger's I

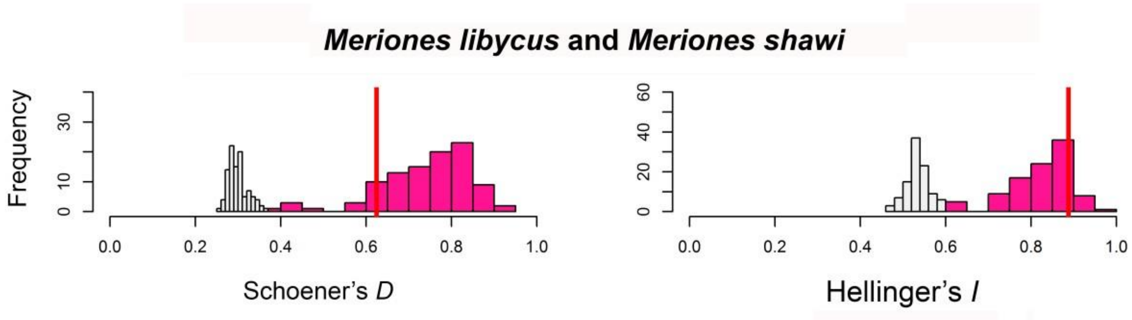

Meriones libycus* and *Meriones shawi
Frequency
0
10
30
0.0
0.2
0.4
0.6
0.8
1.0
Schoener's *D*
0
20
40
60
0.0
0.2
0.4
0.6
0.8
1.0
Hellinger's *I*
