# Supplementary material for: Coarse-resolution Ecology of Etiological Agent, Vector, and Reservoirs of Zoonotic Cutaneous Leishmaniasis in Libya
Source: PLoS Negl Trop Dis. 2016 Feb 10;10(2):e0004381. doi: 10.1371/journal.pntd.0004381 (PMC4749236; doi:10.1371/journal.pntd.0004381)

S6 File: Total annual number of cases reported to the Libyan National Centre for Disease Control 2004-2013. These cases were reported by the local health units in each province and notified to the center for control measures based on the endemic status of each focus. These cases were identified by passive surveillance, and were not diagnosed to the species level.

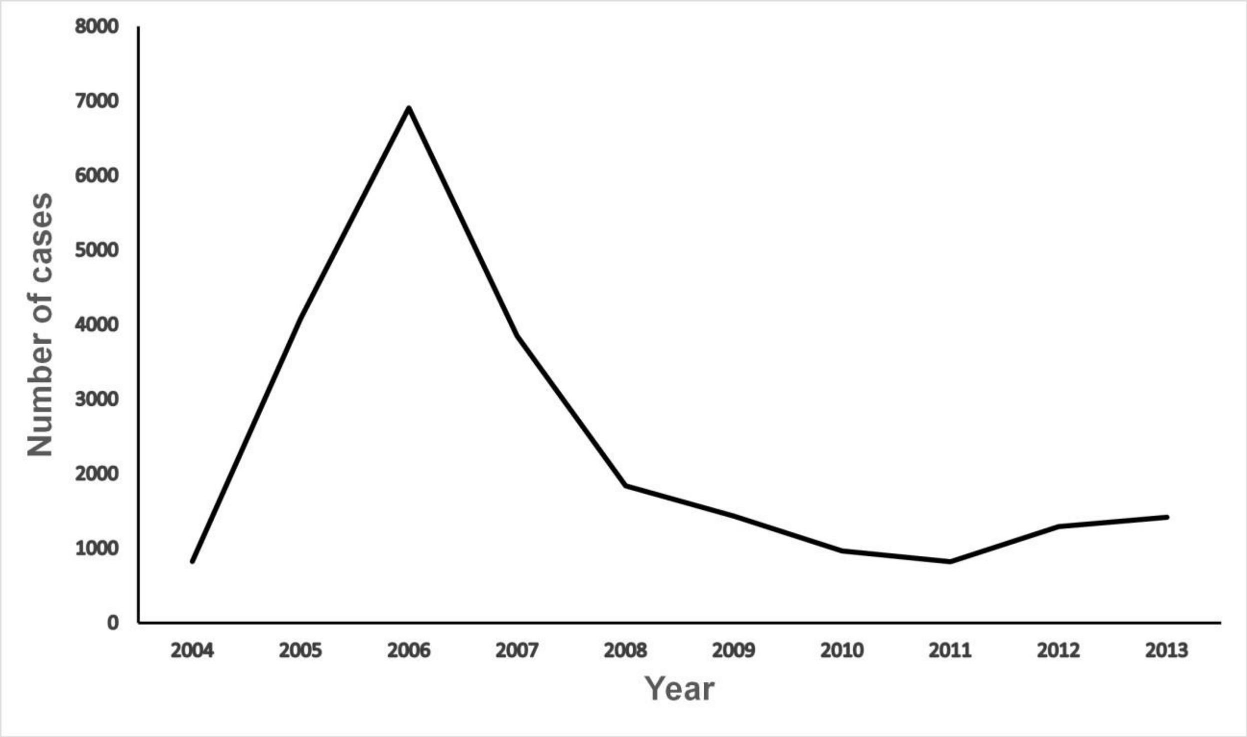

Supplement: S6 File — These cases were reported by the local health units in each province and notified to the center for control measures based on the endemic status of each focus. These cases were identified by passive surveillance, and were not diagnosed to the species level. (PDF) [file pntd.0004381.s006.pdf]
