# Supplementary material for: Coarse-resolution Ecology of Etiological Agent, Vector, and Reservoirs of Zoonotic Cutaneous Leishmaniasis in Libya
Source: PLoS Negl Trop Dis. 2016 Feb 10;10(2):e0004381. doi: 10.1371/journal.pntd.0004381 (PMC4749236; doi:10.1371/journal.pntd.0004381)

S7 File: Localities with high zoonotic cutaneous leishmaniasis incidence and water resource management across Libya. Districts with high incidence are shown in blue, and localities within each district is presented as a dotted points. The map of Libya at the top shows the distribution of areas with water resource management initiatives as blue circles.

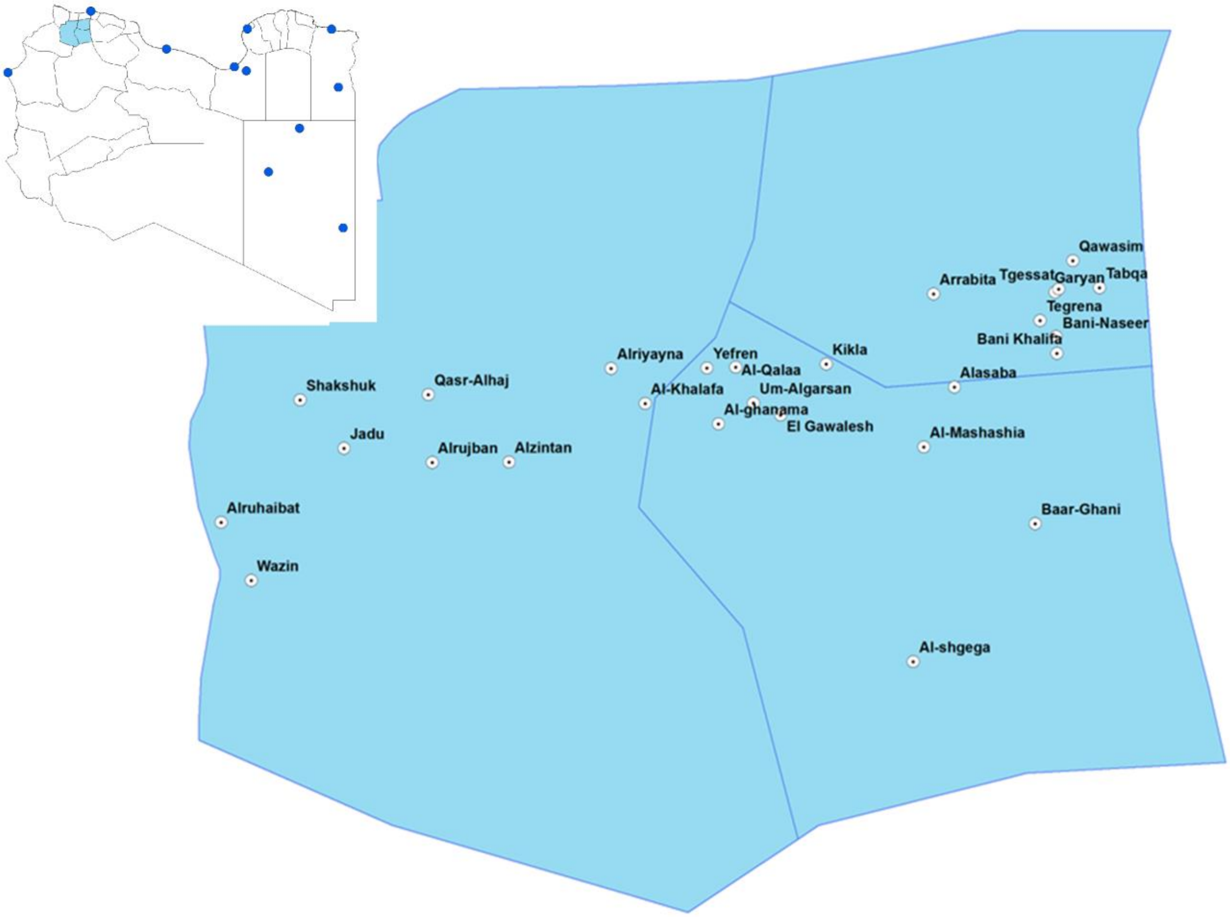

Supplement: S7 File — Districts with high incidence are shown in blue, and localities within each district is presented as a dotted points. The map of Libya at the top shows the distribution of areas with water resource management initiatives as blue circles. (PDF) [file pntd.0004381.s007.pdf]
